# Small fiber neuropathy with long-term, multifocal paresthesias after a SARS-CoV-2 vaccination

**DOI:** 10.1016/j.clinsp.2023.100186

**Published:** 2023-03-13

**Authors:** Josef Finsterer, Fulvio Alexandre Scorza, Carla Alexandra Scorza, Antonio-Carlos G. de Almeida

**Affiliations:** a Neurology & Neurophysiology Center, Vienna, Austria; b Disciplina de Neurociência. Escola Paulista de Medicina, Universidade Federal de São Paulo (UNIFESP/EPM), São Paulo, SP, Brazil; c Centro de Neurociências e Saúde da Mulher “Professor Geraldo Rodrigues de Lima”, Escola Paulista de Medicina, Universidade Federal de São Paulo (EPM/UNIFESP), São Paulo, SP, Brazil

Small fiber neuropathy (SFN) is a peripheral nervous system (PNS) disorder affecting A-delta and C-fibres that conduct in a primarily afferent manner sensory qualities (pin-prick, pain, thermal, mechanical, chemical information) and autonomic information.^1^ SFN usually manifests with sensory disturbances, pain, and autonomic abnormalities.^1^ In general, SFN can be primary (hereditary) or secondary (acquired).^1^ Infectious, metabolic, toxic, immunological paraneoplastic, and neoplastic disorders have been described as causes of acquired SFN.^1^ Among the immunological causes, several vaccines have been identified as the cause of SFN.^2^ There is increasing evidence that SARS-CoV-2 vaccinations can also cause SFN (Table 1).^3^, ^4^, ^5^, ^6^, ^7^, ^8^, ^9^ A patient who developed isolated, multifocal, acral sensory symptoms without pain or autonomic dysfunction for months shortly after SARS-CoV-2 vaccination was not reported.

**Table 1:** Patients with SARS-CoV-2 vaccination-related SFN reported in the literature as per the end of February 2023.

Age (y)	Gender	Brand	Dose	Lat (d)	Symptoms	Reference
52	f	AZV	2.	19	multifocal paresthesias	[index case]
40	f	BPV	2.	10	DN, flushing, diarrhea, MW, GD	^4^
52	f	MOV	2.	17	DN, vertigo, brain fog, palpitations, dysphagia, insomnia	^4^
32	f	MOV	2.	1	fatigue, brain fog, palpitations, vertigo, MW, SD, hives	^4^
12 pat.	nr	nr	nr	nr	POTS, hypohidrosis, tachycardia, Raynaud, SD	^5^
43	m	BPV	1.	3	nr	^6^
39	m	BPV	1.	10	pain, numbness, tingling, fasciculations, dysesthesia	^7^
60s	f	AZV	1.	7	dysesthesias, hypoalgesia, low vibration	^8^
50s	f	AZV	1.	10	dysesthesias, thermhypesthesia	^8^
60s	m	AZV	1.	15	dysesthesia, thermhypesthesia	^8^
27	f	BPV	2.	Nr	MW, neuropathy, fatigue, neuralgia, paresthesia.	^9^
57	f	BPV	2.	7	burning dysesthesias	^10^
52	m	BPV	2.	Nr	paresthesias, burning and stabbing pain, tinnitus	^11^

AZV, Astra Zeneca vaccine; BPV, Biontec Pfizer vaccine; d, days, DN, dizziness; GD, gait disturbance; Lat, latency between vaccination and onset of SFN; MOV, Moderna vaccine; MW, muscle weakness; POTS, postural tachycardia syndrome; SD, sensory disturbances; y, years.

The patient is a 52 years-old female, diagnosed with SARS-CoV-2 vaccine-related SFN based on history, clinical presentation, nerve conduction studies (NCSs), and skin biopsy from two sites. She presented 12 days after the second dose of the Astra Zeneca vaccine (AZV) with paresthesias affecting the tongue, lips, all fingers, and all toes. She did not complain of pain or any autonomic symptoms. Her history was positive for tinnitus, left-side glaucoma, right lower limb fractures due to trauma, menstrual problems, and bee and wasp allergy. Her individual and family history was negative for acquired or hereditary disorders of the peripheral nervous system. She didn't take any medication regularly.

The clinical-neurologic exam revealed only sore neck muscles. There was no hypoesthesia, dysesthesia, or allodynia in the area of the paresthesias. Blood tests showed only a moderately reduced glomerular filtration rate (66.0 ml/min (n: > 90 ml/min)). NCSs revealed only an axonal lesion of the right peroneal nerve, which was attributed to multiple fractures of the right lower limb. Magnetic resonance imaging (MRI) of the entire spine showed only osteochondrosis C5-C7. Lumbar puncture was not informative. A skin biopsy of the left thigh and the left lower leg revealed a marked reduction of the intra-epidermal nerve fiber density (IENFD) to 1.75 +/-0.66 (n, 9.8-10.8)^12^ at the distal lower limb and to 2.83 +/- 0.14 (n, 10.6-31.4)^13^ at the thigh. Extensive screening for secondary causes of SFN in the serum and urine was all inconclusive. Computed tomography (CT) of the abdomen showed only a single cortical renal cyst. The patient was suggested to take pregabalin but she did not agree. At the seven-month follow-up, she reported a slight reduction in her paresthesias without taking any medication.

This case shows that SARS-CoV-2 vaccinations can have side effects even without significant comorbidities, that SARS-CoV-2 vaccinations can cause SFN without pain or autonomic symptoms, that these symptoms can persist for months, and that they decrease without symptomatic treatment over time.

Since the introduction of anti-SARS-CoV-2 vaccines evidence has accumulated that none of the commercially available vaccines is free of side effects for everyone. Most commonly, side effects occur in the central and peripheral nervous systems.^14^ One of the side effects affecting the PNS is SFN. SFN due to SARS-CoV-2 vaccines is increasingly recognized and can occur as pure SFN^8^ or in combination with other neurological or non-neurological side effects.^4^ Common symptoms of SARS-CoV-2 vaccination-associated SFN are hypoesthesia, thermohypoesthesia, paresthesia, dysesthesia, reduced vibration sense and focal, regional, or whole-body pain.^4^, ^5^, ^6^, ^7^, ^8^ In addition to sensory disturbances, these patients may develop autonomic abnormalities such as tachycardia, postural tachycardia syndrome (POTS), diarrhea, orthostasis, heat intolerance, hypohidrosis, and palpitations.^5^ Diagnosing SFN is based on history, clinical exams, blood tests, nerve conduction studies, and skin biopsies. The gold standard for diagnosing SFN is documentation of reduced IENFD or reduced sweat gland nerve fiber density (SGNFD).^1^ The reduction of small fiber density can also be seen in corneal confocal microscopy and other techniques. There is no general agreement on how to treat SARS-CoV-2 vaccination-related SFN, but according to the guidelines for the treatment of SFN in general, patients with SARS-CoV-2 vaccination-related SFN can benefit on the one hand, from symptomatic measures against pain, dysesthesias, allodynia, autonomic dysfunction^9^ and on the other hand from immunosuppressive treatment using glucocorticoids and intravenous immunoglobulins (IVIGs).^5^

In summary, this case shows that SARS-CoV-2 vaccines are not safe for everyone and that their side effects can last for months, and can lead to long-term impairment. There is a need to produce safer anti-SARS-CoV-2 vaccines in case the pandemic recurs.

## Funding

No funding was received.

## Data access statement

All data are available from the corresponding author.

## Authors’ contributions

JF: design, literature search, discussion, first draft, critical comments, final approval.

## Data access statement

Not applicable.

## Compliance with ethics guidelines

This article is based on previously conducted studies and does not contain any new studies with human participants or animals performed by any of the authors.

## Conflicts of interest

The authors declare no conflicts of interest.
